# Linear Magnetization Curve with Extremely Low Permeability Obtained via Stress Annealing of Fe- and Co-Based Nanocrystalline Alloys

**DOI:** 10.3390/ma19050844

**Published:** 2026-02-25

**Authors:** Otto K. Temesi, Albert Karacs, Gábor Gulyás, Sándor Komáromi, Lajos K. Varga

**Affiliations:** 1 H-ION Kft., H-1121 Budapest, Hungary; otto.temesi@h-ion.hu (O.K.T.); gabor.gulyas@h-ion.hu (G.G.); 2 Mikro-T Kft., H-1121 Budapest, Hungary; karacsal@mikrot.hu; 3 Progen Kft., H-1118 Budapest, Hungary; komaromi@progenkfi.com; 4 Institute for Solid State Physics and Optics, Wigner Research Center for Physics, H-1525 Budapest, Hungary

**Keywords:** metal-amorphous nanocomposite, MANC, Fe- and Co-based MANCs, stress-induced anisotropy, magneto-crystalline and magneto-strictive energy, Herzer model, measure of linearity, linear magnetization, small permeability

## Abstract

First, the properties of the linear magnetizing curve and low static permeability are summarized. Second, a design for technical implementation of mechanical stress-induced anisotropy in metal-amorphous nanocomposites (MANCs) is presented. Stress annealing, which creates the conditions for a linear magnetizing curve, is an order of magnitude more effective with Co-based MANCs than with Fe-based ones. Permeabilities between 3000 and 100 and between 100 and 10 can be obtained in Fe- and Co-based nanocomposites, respectively, at similar applied tensile stresses. A measure of linearity is introduced based on the parameters of the hysteresis loop, which is proven to be equal to the fraction of the crystalline phase responsible for the induced anisotropy. Lastly, experimental results concerning linearity and related properties are discussed.

## 1. Introduction

### 1.1. Generalities

The global electricity consumption in 2022 was 24,398 terawatt–hours (TWh), almost exactly three times the consumption in 1981 (8132 TWh) [1], and it is safe to say that the importance of soft magnetic materials research has increased proportionally. There is one topic that has not received attention commensurate with its importance in the design and production of soft magnetic components: manipulation of the magnetization curve by creating transverse anisotropy and tailoring its properties according to the requirements.

The magnetization curve is necessarily a saturation curve with a linear section only spanning up to a certain fraction of the saturation value. The larger this section, the more suitable it is for various applications.

**Figure 1:**
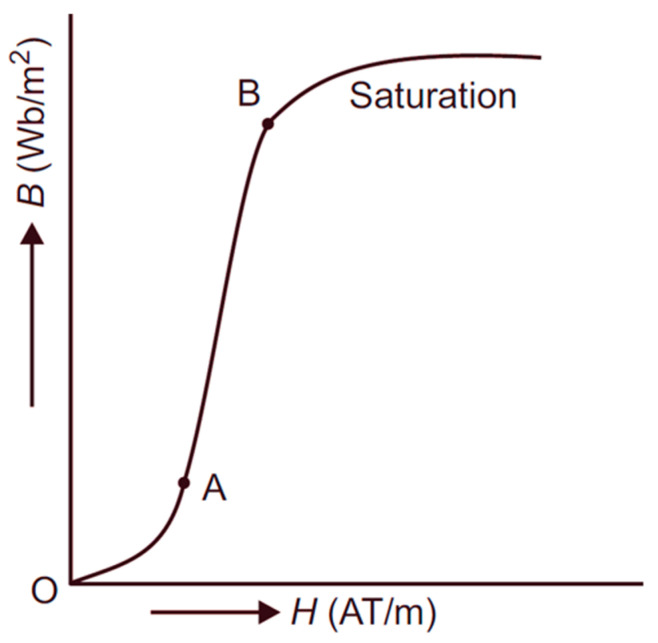
Illustration of linear portion of the magnetization curve between the ankle (A) and knee (B) points.

In general, based on domain wall movement magnetization, there are two curvatures on the magnetization curve (see Figure 1): at a small field characterized by the ankle point, A, the end of the Rayleigh region, and at large fields, the knee point, B, near the start of the saturation. The linear section lies between these two curved regions. By creating the conditions for domain rotation magnetization via transversal-induced anisotropy, the ankle point can be moved to the origin, thus enlarging the linear portion.

The linear portion of the magnetization curve is assessed to accurately model performance, calculate energy losses, and ensure efficient operation in devices such as transformers, inductors, and motors. It also provides an advantage during applications; for example, linear B-H magnetization is necessary to maintain constant permeability in the case of randomly appearing DC components in filter applications.

The benefit of low permeability is that the stored magnetic energy is greater at a lower effective permeability. The magnetic energy stored in a soft magnetic material can be calculated as follows:

E = B^2^V/(2µ_o_µ) (1)

where B is the induced flux density, V is the core volume, µ is the relative permeability, and µ_o_ is a universal constant; i.e., the permeability of vacuum (µ_o_ = 4π10^−7^ Vs/Am).

The stored power will be

(2) $$\frac{P_{st}}{V}=f\frac{B_{m}^{2}}{2\mu \mu_{o}}$$

A primary source of losses is from classical eddy currents. This power loss can be expressed by

(3) $$P_{e}=cf^{2}B^{2}$$

where the pre-factor c is

(4) $$c=\frac{{\left(\pi \cdot t\right)}^{2}}{\rho }$$

In Equation (3), f is frequency and B is induction; in Equation (4), t is thickness and ρ is resistivity.

The core loss at a higher frequency and near saturation magnetization is given by the Steinmetz [2] formula:

(5) $$\frac{P_{core}}{V}=k_{s}f^{\alpha }B_{m}^{\beta }$$

where k_s_, α, and β are parameters to be fitted, which range from 1 to 1.4, 1.12 to 1.21, and 1.8 to 2.1, respectively.

Concerning the static hysteresis, the Steinmetz formula is simplified as [2,3]:

*Pcore/V* = *K_DC_*·*B^n^* (6)

Expansion of the Taylor series of the Rayleigh relation [4] defines the Steinmetz coefficient as n = 3, indicating that the Steinmetz formula is valid only on the linear section of the magnetic curve [2,3]: 0.3 T ≤ B_i_ ≤ 1.2 T.

The largest possible storage energy and the independence of permeability from the DC bias current can be achieved if the relative permeability is as small as possible and the hysteresis curve is as linear as possible up to magnetic saturation. It is a self-evident condition that the magnetic field associated with the linear section should be larger than the field of randomly appearing DC currents of any origin, which could possibly push the permeability of the core out of its operating range. However, a sufficiently large magnetization level is only achieved with a sufficiently large copper wire winding; this, in turn, increases the copper loss.

(7) $$P_{w}=RI^{2}$$

The minimum iron and copper losses are achieved in the case of a toroidal iron core of length l, volume V, number of turns N, and copper resistance R for the following relative permeability:

(8) $$\mu_{opt}=\frac{l}{N}\sqrt{\frac{R}{2k_{s}Vf^{1/2}}}$$

Therefore, low permeability is required for operation at high frequencies, while high permeability is required for low copper loss. The task of the design engineer in this regard is optimization; for this purpose, an iron core with a designable permeability that satisfies the optimum condition is required.

**Figure 2:**
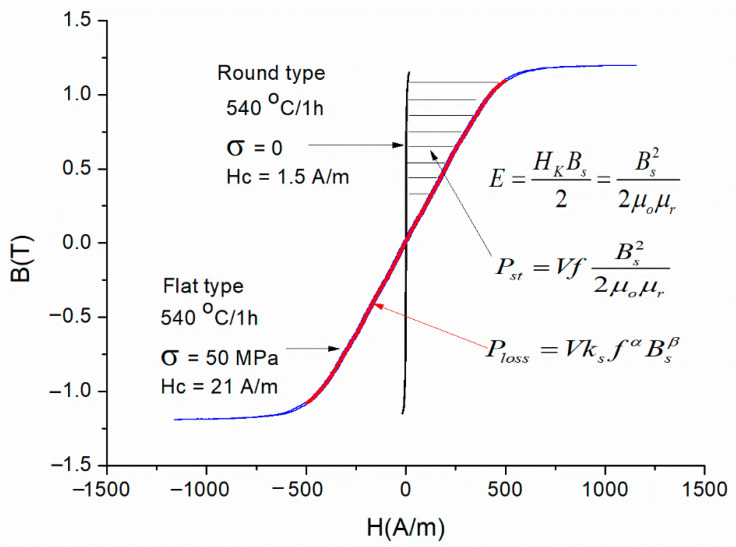
Illustration of stored energy, stored power, and power loss on a core that was heat-treated with and without mechanical stress.

The stored energy (see Figure 2) can be expressed using the characteristics of magnetic material, and at the same time, as inductive energy:

(9) $$\frac{L\cdot I_{p}^{2}}{2}=\frac{B_{p}^{2}}{2\cdot \mu_{o}\cdot \mu_{eff}}\cdot V$$

Comparing these two expressions of stored energies it turns out that, for the same stored energy, the volume of the core must decrease along with the permeability of the core:


(10) $$\frac{\mu_{eff}}{V}=\frac{B_{p}^{2}}{\mu_{o}\cdot L\cdot I_{p}^{2}}$$

Unfortunately, the majority of heat generated in the magnetic circuit stems from the storage function, and the task of “material by design” is to find the inductive material that stores the most energy with the lowest core and winding losses. The performance or quality factor of the material can be expressed as the ratio of stored power to power loss:


(11) $$P_{ratio}=\frac{P_{stored}}{P_{loss}}=\frac{\frac{B_{m}^{2}}{2\mu_{o}\mu_{r}}f}{k_{S}f^{\alpha }B_{m}^{\beta }}=\frac{B_{m}^{2-\beta }f^{1-\alpha }}{2k_{S}\mu_{o}\mu_{r}}$$

It can be seen that the lower the permeability, the higher the frequency at which a quality factor greater than 10 can be achieved. We draw attention to the fact that for Equation (11), technical units must be used (*f* in kHz, *P_core_/V* in mW/cm^3^, *B* in Tesla, *µ_r_* in numbers) in order to obtain a valid performance ratio:

Example for power loss calculations:

Let *µ_r_* = 1000, f = 1.5 kHz, B = 0.2 T, Ks = 3.935, α = 1.585, β = 1.888;

then, $P_{core}$/V = 3.935 × 1.5^1.585^ × 2^1.888^ = 3.935 × 1.9 × 3.7 = 2.66 mW/cm^3^ = 3.68 W/kg.

At 100 kHz, 100^1.585^ = 1479, and keep the magnetization at 0.2 T.

$P_{core}$/V = 3.935 × 1479 × 0.0479= 278.77 mW/cm^3^ = 37.17 W/kg.

Example for $P_{ratio}$ calculations:

Let f = 10 kHz, µ = 1000, B = 0.1;

then, P_ratio_ = 20.

It seems that it is not difficult to establish parameters corresponding to a P_ratio_ > 10.

More details on the performance ratio can be found in [5].

### 1.2. Description of Linearity

It is most advantageous to represent the linear magnetization with an anhysteretic curve, in which the lower curvature is absent and the section of the saturating curvature is as short as possible. Mathematically, many such functions can be used; however, in practice, the following three are the most widespread: Langevin Equation (12), T model Equation (13), and a rational expression used by powder iron core-producing companies Equation (14).

L(x) = coth(x) – 1/x(12)

T(x) = tanh(x)(13)

(14) $$B=\frac{\mu_{\max}}{\frac{1}{H+a\cdot H^{b}}+\frac{1}{c\cdot H^{d}}+\frac{1}{e}}$$

The Langevin function [6] refers to isotropic materials where the individual noninteracting magnetic moments are subjected to the ordering effect of Zeeman energy and to the disordering effect of thermal energy. The latter term can be replaced by the disordering effect of randomly distributed local anisotropy fields H_K_:

(15) $$x=\frac{m_{p}H}{m_{p}H_{K}}=\frac{H}{H_{K}}$$

where *m_p_* is the magnetic moment of particles.

The magnetization to be represented in normalized form (m versus H/T) will be

(16) $$m=\frac{M}{M_{s}}=L\left(\frac{m_{p}H}{k_{B}T}\right)=\coth\left(\frac{m_{p}H}{k_{B}T}\right)-\left(\frac{k_{B}T}{m_{p}H}\right)$$

Most likely, the first application of the Langevin function beyond its original purpose for para- and super-paramagnetic particles was performed using the Jiles–Atherton model [7], where the independent variable x = *m_p_H*/*k_B_T* was rewritten as *x* = *H*/*a*, with *a* being one of the key parameters of the J-A model. At a higher level of hysteresis modeling, it was recognized that the disordering effect of thermal energy can be replaced by the disordering effect of a randomly distributed local anisotropy field, H_K_, against which the rotation of magnetization occurs during the magnetization process. Such local anisotropy can be created, for example, by a demagnetizing field of powder particles of differing sizes and forms. The new variable will be *x* = *H*/*H_K_*, and the magnetization is calculated using Equations (17) and (18):

*M* (*H*) = *m_p_*·*N*·*L*(*x*), (17)

where *L*(*x*) = *coth*(*H*/*H_K_*) − *H_K_*/*H* (18)

The T(x) model [8] is based on the tanh(x) saturating function. It seems to be mathematically easier to work with than the *L*(*x*) function. The magnetization will be given by an expression similar to Equation (17):

*M*(*H*) = *m_p_*·*N*·*tanh*(*x*) (19)

The rational function model [9,10] is mostly used by companies to provide the *M*(*H*) curves of their powder core products. In the last two cases, the variable can be the magnetizing field *H* and, in normalized form, Equation (18), where *H_K_* is the mean value of the local anisotropy field distribution, *p*(*H_K_*), obtainable via a double derivative of the upper remanent part of the hysteresis loop [11,12].

H_K_ can be calculated using the effective permeability as well:

*H_K_* = *B_s_*/*µ_o_µ* (20)

One can conclude that, in order to realize the largest linear portion, we have to create uniaxial anisotropy, which defines the easy axis of magnetization parallel to the toroid core axis. In this case, the magnetizing field is pointed to the hard axis of magnetization and the magnetization occurs via rotation, which occurs with zero coercivity and a missing ankle point:

*B*(*H*) = (*H*/*H_K_*) · *B_s_* (21)

We are aware of three technologies used to induce uniaxial anisotropy: 1. shape anisotropy, 2. annealing in a transversal magnetic field, and 3. annealing under tensile stress. The first requires the cutting of a special geometrical form before nano-crystallization heat treatment. It exhibits the lowest productivity. For transversal magnetic field annealing, one must adapt the composition of a Finemet-type alloy to the desired range of the obtainable permeability interval. The following permeability data ranges are covered using Fe-based MANCs with different trade names:

- Normal Finemet (Vitroperm 500 F): 17,000–100,000;
- Doped Finemet (with Ni) (Vitroperm 250F): 3000–5000;
- Doped Finemet (with Ni and Co) (Vitroperm 712): 12,000.

Although magnetic field annealing is the most productive, cores with small permeabilities cannot be prepared. Meanwhile, despite the low productivity of stress annealing, a truly flat magnetization curve can be obtained at more advantageous costs.

In general, the induced anisotropy can be applied to both amorphous and crystalline materials. The present subject, however, is restricted to Fe- and Co-based metal-amorphous nanocomposites (MANCs) consisting of a 75–80 percent volume of nanosized crystalline precipitate embedded in a 20–25 percent volume of the residual amorphous phase. The starting material is an amorphous ribbon permitting two-stage crystallization which is well separated in the temperature domain.

In the Fe-based nanocomposite (called Finemet alloy: Fe_73.5_Si_15.5_B_7_Nb_3_Cu_1_), Fe_80_Si_20_ is the composition of the crystalline phase, with a magneto-crystalline anisotropy energy of E_MCA_ = 8000 J/m^3^ and a magneto-strictive coefficient of about λ = −9 ppm. At the same time, in the Co-based nanocomposite, a “zero lambda alloy,” (Co_95_Fe_5_)_78_Nb_5_Si_2_B_14_Cu_1_ is the nanoprecipitate that consists of pure or solid-solution hcp Co with magneto-crystalline anisotropies (K_1_ = 4.5 × 10^5^ and K_2_ = 1.5 × 10^5^ J/m^3^) that are orders of magnitude larger than that of Fe_80_Si_20_ (K_1_ = 8000 J/m^3^). The Co-based material presents two allotropic phases: the stable hcp phase below 330 °C and a metastable fcc phase above 440 °C. The group at KTH Stockholm, led by Prof. L. Vitos, performed ab initio (DFT) calculations [13] concerning the allotrope phase transition of Cobalt. They found that the hcp phase is the ground state and the energy difference between the hcp and fcc phases is about 15 mRy at T = 0 K; they also found that vibrations of the ionic lattice make the largest energy contribution to the destabilization of the hcp phase. The calculated transition temperature, 825 K, is a slight overestimation of the experimental temperature of 713 K. The E_MCA_ of λ hcp Co is found to be two orders of magnitude larger than that of fcc Co. For hcp Co, <0001> is the lowest packed direction and is the easy axis. Meanwhile, <1000> is the close-packed direction, corresponding to the hard axis.

Thus, the induced anisotropy creates a hard axis, and magnetization along the hard axis produces a perfect linear magnetization ($B=B_{s}\cdot H/H_{s}$), where the ankle point is missing and coercivity is negligible.

The easy axis’ magnetization describes a hysteresis loop with $H_{c}\;=\;2K_{u}/B_{s}$, which is equal to the anisotropy field $H_{a}$ in the ideal case.

The saturation magnetostriction [14] is a two-constant (λ111 and λ100) equation for cubic crystals and a four-constant (λA, λB, λC, and λD) equation for hcp crystals. This reduces to two constants when the magnetostriction is measured in the same direction as the magnetization. For the hcp cobalt monocrystal, the value along the c axis is λ100 = −140 ppm and, perpendicular to the c axis, it is λ111 = +50 ppm. From these constants obtained for monocrystals, the value for polycrystal is calculated as a function of symmetry-determined angular dependence: $\lambda_{s}$ = −62 ppm. The stress-induced magneto-elastic energy will be proportional to the volume fraction of crystalline phase and the applied stress:


(22) $$E_{me}=-\frac{3}{2}\lambda_{s}\cdot v_{cr}\cdot \sigma$$

For Fe-based nanocomposites, the conditions are clearly defined: $\lambda_{s}$ = −9 ppm; the volume fraction of Fe_80_Si_20_ nanophase is about 0.75, so it is possible to plan the extent of induced anisotropy:

E_me_ = Ku ≅ (10 ÷ 11) · σ. (23)

For Co, this is not straightforward as the nanoparticles present an angular distribution and a partial fcc–hcp transformation. Based on the Co monocrystal data, the maximal induced anisotropy will be

E_me_ = Ku ≅ 157 · σ. (24a)

Based on the Co polycrystal data, the maximal induced anisotropy will be

E_me_ = Ku ≅ 62 · σ. (24b)

Next, we present the production technology and evaluation method for stress annealing, checking Equations (23) and (24) based on our results, as well as numerical results derived from the literature. This procedure permits the desired linearity and small effective permeability to be obtained.

## 2. Experimental Design

The scope is to fill the permeability gap between powder cores (µ ~ 10–200) and magnetic field annealing (µ ~ 3000–100,000). The homogeneity of the produced nanocrystalline composite structure ensures low coercive force (less than 10 A/m) even at the highest applied tensile stress, when the induced anisotropy reaches 12,000 J/m^3^ for Fe-based and 70,000 J/m^3^ for Co-based MANCs. This allows the magnetization to increase linearly up to the theoretical limit of 70–72%. By varying the tensile stress, the effective permeability can vary between 100 and 3000 for Fe-based MANCs and between 10 and 100 for Co-based MANCs, which allows the cut-off frequency of the inductance operation to vary between 2 and 40 MHz, while the permeability and iron loss change negligibly between –20 and 150 °C. The working temperature can reach 250 °C without any deterioration of the magnetic parameters over time. These technical parameters, as well as the moderate saturation induction (1.23 T), enable the widespread use of stress-annealed MANC cores in high-frequency and high-temperature power electronics applications.

**Figure 3:**
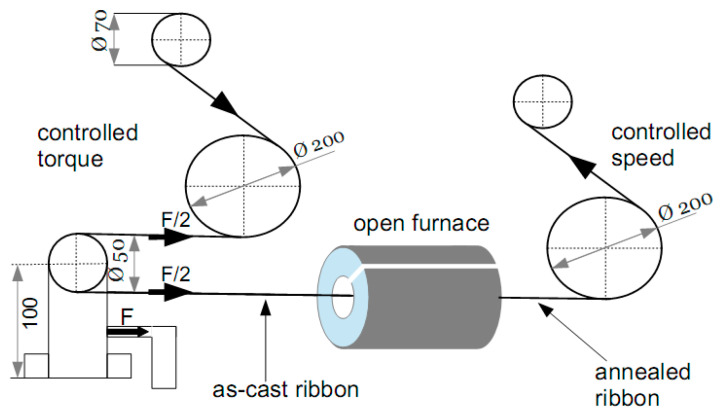
Sketch of the home-built continuous stress annealing equipment.

Acceptable productivity can be obtained via continuous stress annealing alone, without protective gas. A schematic drawing of the designed quench-up equipment is presented in Figure 3. The particular feature of this technique is the cutting of the tubular furnace along its generator, permitting placement of the running amorphous ribbon into the hot cylinder. A constant pulling stress between 10 and 500 MPa is applied on the ribbon running reel-to-reel, with a constant velocity between 0.1 and 1 m/s. The pulling force is kept constant through feedback to the two servo-motors, and its value F is measured with a dynamometer. The elongation or contraction of the ribbon can be determined by reading the position of the motors through an incremental encoder. The contraction might appear when the negative strain due to the amorphous–crystalline transformation is larger than the elongation due to creep.

For mass production, only continuous pulling through a tubular furnace in air should be considered. For continuous stress annealing, a high-quality amorphous ribbon should be applied. The ribbon with a thickness of 18–25 µm and a width of 1–4 mm is conducted through a tubular furnace heated using an appropriate number of halogen lamps, which produces a special longitudinal thermal gradient between 25 and 700 °C. The nanocrystalline ribbon produced during the stress-induced heat treatment is brittle but has sufficient flexibility to be rewound from the large, nano-crystallized ribbon storage disk to the casing of the small iron cores. During the heat treatment in open air, an insulating surface layer is formed that provides adequate insulation between the nanocrystalline strip layers, which enables the production of large cores in one step by winding the nanocrystalline strip onto the final core coil instead of the reservoir disk. By appropriately adjusting the heat treatment parameters, a narrow distribution of nanosized Fe-Si precipitates can be achieved, which ensures the adequate flexibility of the heat-treated strip. If the minimum bending radius without breakage is used to characterize the flexibility, the bending radius of the nanocrystalline strip produced by our equipment reaches 1–2 mm, even after heat treatment under the highest stress (500 mPa), which allows for safe handling of the heat-treated ribbon.

## 3. Evaluation of Stress Annealing

Evaluation of the Fe- and Co-based nanocomposites was performed based on Herzer’s back-stress theory [15,16]. The stress applied during flash annealing will be preserved by the residual amorphous phase. During stress annealing, an inelastic creep deformation of the residual amorphous matrix occurs, resulting in post-cooling residual stress acting on crystallites. This residual stress (σ), in combination with the magnetostriction constant of (Fe_80_Si_20_ or hcp Co-based solid solution) nanoprecipitates, gives rise to the induced anisotropy (Equation (22)):

(25) $$K_{ind}=-\frac{3}{2}\lambda_{cr}^{s}x_{cr}\sigma$$

where $\lambda_{cr}^{s}$ is the saturating magnetostriction coefficient of the crystalline phase and $x_{cr}$ is the volume concentration fraction of the crystalline phase.

It is assumed that the internal residual stress is equal to the externally applied stress during annealing. The induced anisotropy can be expressed with the help of permeability as well as the semi-product of the anisotropy field (Equation (20)) and the saturation magnetization, B_s_:

(26) $$K_{ind}=\frac{B_{s}^{2}}{2\mu_{o}\mu }$$

There is experimental evidence [15,16] that the permeability of a flattened linear magnetization curve is inversely proportional to the applied stress; their product is constant:

(27a) μ⋅σ=53850 for Fe-based

(27b) μ⋅σ=2500÷5700 for Co-based

where σ is given in MPa.

When the permeability and stress are known, using Equations (25)–(27), the product of the unknown magnetostriction and volume fraction can be determined as

(28a) $$\lambda_{cr}^{s}x_{cr}=\frac{B_{s}^{2}}{3\cdot (\mu \sigma )}$$

When the induced anisotropy and the applied stress are known, the unknown product of the crystalline fraction and its magneto-elastic constant can be determined as

(28b) $$\lambda_{cr}^{s}x_{cr}=\frac{2}{3}\frac{K_{u}}{\sigma }$$

It turns out that the unknown product, $\lambda_{cr}^{s}x_{cr}$, can be determined either from the preparation parameter, σ, and hysteresis loop parameter, µ, via Equation (28a) or Equation (28b), determining the k = Ku/ σ parameters from the hysteresis loop.

For Fe-based MANCs, Herzer [16] determined the magnetostriction coefficient for the Fe-Si nanoprecipitate: $\lambda_{cr}^{s}$ = −9 ÷ −10 ppm. Hungarian authors [17,18] have demonstrated that the effective permeability does not depend on the ribbon elongation, just on the applied stress.

**Figure 4:**
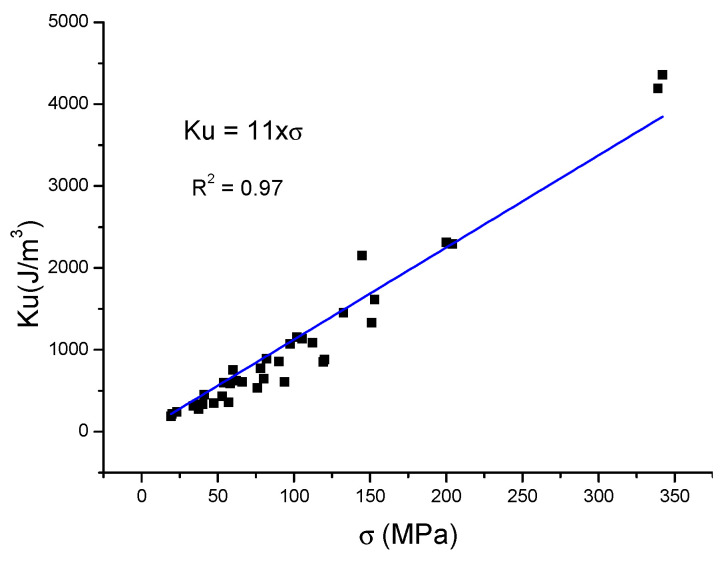
Stress-induced anisotropy, Ku, of Fe-based MANCs.

**Figure 5:**
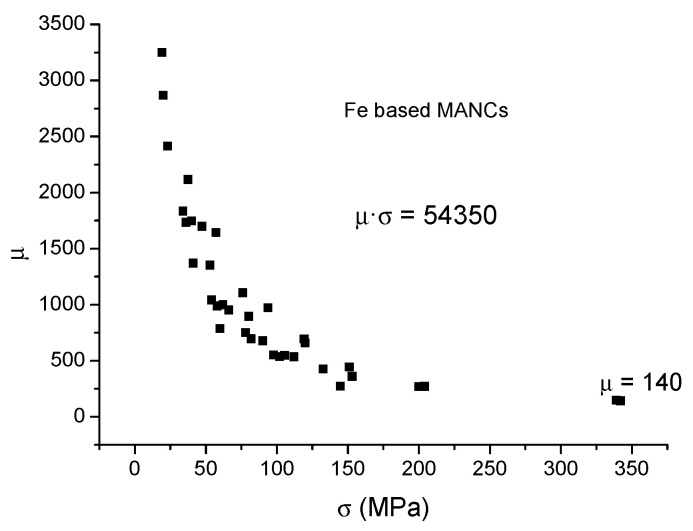
Permeability values, µ, obtainable via stress annealing of Fe-based MANCs.

Collected stress-induced anisotropy and effective permeability data for Fe-based MANCHs [19,20,21] are shown in Figure 4 and Figure 5, respectively. From the fitting of the data, the average stress sensitivity was determined: Ku/ σ = 11 and µ = 54,350/σ.

Taking Equation (23), $\lambda_{cr}^{s}x_{cr}$ ≅ (2/3) × 11, and accepting the above values for $\lambda_{cr}^{s}$, the fraction of the crystalline phase is approximately x_cr_ = (2/3) × (11/10) = 0.73. This is the optimal fraction of the crystalline phase in order to compensate for the positive contribution of the residual amorphous phase to the resultant λ_total_ = 0.73 × (−10) + 0.27 × (28) = −7.3 + 7.56 = 0.26 ppm. The time–temperature product value of heat treatment should be properly chosen to avoid under- or overdone heat treatments, which are marked by a crystalline fraction that is smaller or higher than the optimum.

For Co-based MANCs, we must recall the specific properties of Co:

The maximal magneto-elastic-induced anisotropy is given by Equation (24a,b), depending on whether uniaxial or random-oriented nanosized monocrystals are considered nanosized precipitates. A uniaxial orientation can be approached by annealing under tensile stress. In general, under mass production conditions, poly-crystalline-like random orientation dominates.

The maximal induced magneto-strictive anisotropy is as follows:

(29a) $$K_{ind}=-\frac{3}{2}\lambda_{cr}^{s}x_{cr}\sigma$$

where λ_hcp_ ≅ −62 ppm and x_cr_ ≅ 0.75 ÷ 0.8.

Thus, the maximal induced anisotropy will be 45 kJ/m^3^, supposing that the maximal available stress under mass-production conditions is 600 MPa:

Ku = (70 ÷ 75) × σ (29b)

**Figure 6:**
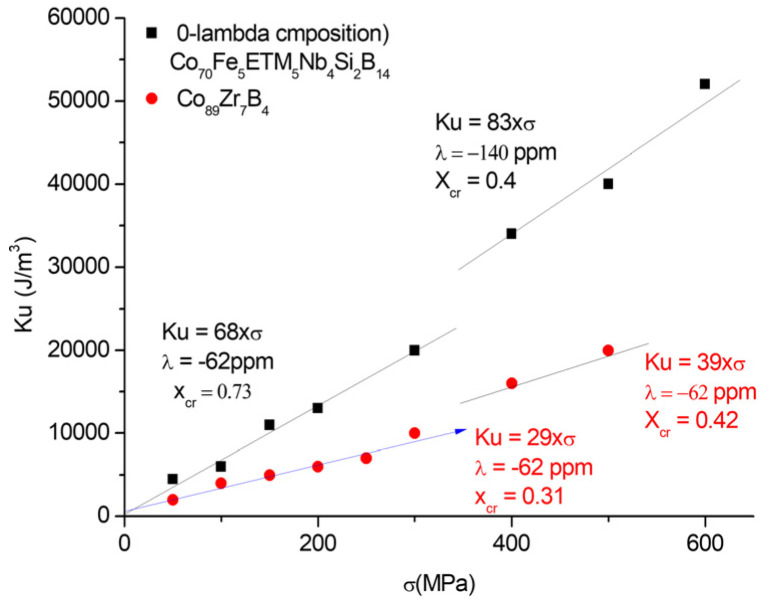
Stress-induced anisotropy in Co-based MANCs.

**Figure 7:**
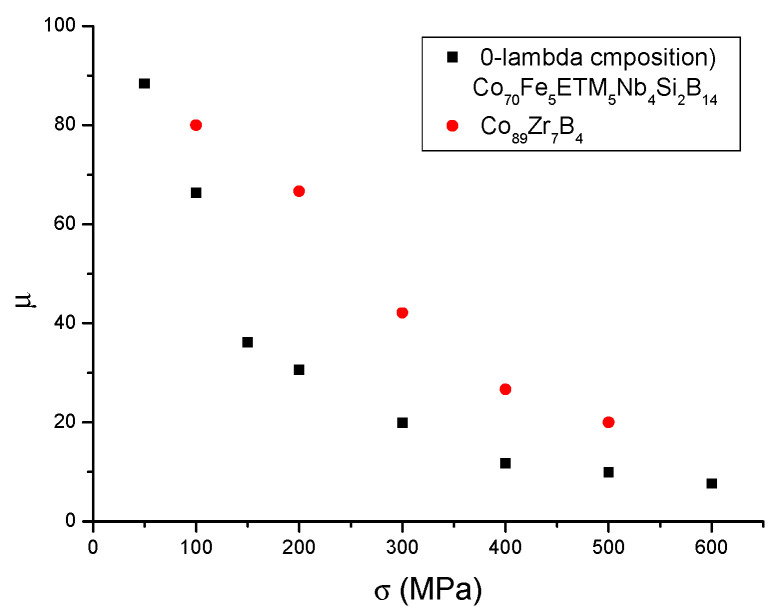
Record-small permeability of stress-annealed Co-based MANCs.

Next, we review the method of producing inductive cores based on Co-based MANCs operating in the frequency range of 1–100 MHz. The precursor amorphous ribbon is a homogeneous 4–25 mm wide ribbon produced during a rapid cooling process, with a thickness of 20–30 μm. This ribbon will be heat-treated under tensile stress to form a Co-based Finemet-type nanocrystalline ribbon from which the desired core size can be wound. In the following, we summarize the currently available known processes. The most important articles [22,23,24,25] are from the McHenry group (Carnegie Mellon Univ., Pittsburgh), who discovered that a huge strain-induced anisotropy can be created in the Co-based Finemet-type alloy, as shown in Figure 6 and Figure 7. Using the same amorphous-forming additive (Nb_4_Si_2_B_14_), they changed the doping elements (Fe_4_Mn_4_, Fe_4_Cr_4_, Fe_5_V_5_) at the expense of the Co content. They found that the earlier the doping (with elements on the left), the higher the possible induced anisotropy. From this, they concluded that the early transition metal dopants promote HCP transformation, which causes the large induced anisotropy. If the amorphous-forming elements contain Zr (e.g., Zr_7_B_4_)—which cannot be prepared in air—the Co nanoprecipitate shows only a small tendency toward HCP transformation, and this tendency is not promoted by the Fe additive. The anisotropy energy that can be induced in the V-doped Co-based MANCs (70 kJ/m^3^ under a stress of 500 MPa) is comparable to the energy stored in the bonded Nd magnet (Nd2Fe14B) (60–100 kJ/m^3^). When translated into the language of permeability, the figure below clearly shows that in practice, with easily achievable stresses of 200–300 MPa, the effective permeability can be reduced to values between 20 and 60.

Cobalt-rich amorphous alloys, with a Co/Fe ratio of about 95/5, have very low (often near-zero) magnetostriction (λ ≈ 0 to ±3 × 10^−6^), representing the so-called zero lambda alloys with extremely good soft magnetic properties. A small lambda results in a small magneto-elastic energy term when stress σ is applied during annealing, which induces an easy (or hard) axis of magnetization, depending on the sign of λ.

Much larger anisotropy is obtained in the nanocrystalline state, when the magneto-crystalline anisotropies of hcp Cobalt are present due to the precipitation and phase transformation of nanophase Cobalt. The precipitation of hcp instead of fcc Cobalt is catalyzed by the addition of 1–5 at% early transition elements. The applied stress during the heat treatment also facilitates the fcc–hcp phase transformation. However, this transformation is not completed during the flash annealing that occurs during the process of pulling through the tubular furnace, which is a short-term and high-temperature treatment. This is why a long-term heat treatment should follow at 603 K (about 330 °C), thus helping to complete the fcc-bcc transformation. It is worth mentioning that the hcp phase is stable up to 690 K (417 °C); these data are valid for the bulk Cobalt. The hcp-to-fcc transition temperature increases up to 800 °C when nanoclusters of about 5 nm are considered. The Co nanoprecipitates with random angular orientation behave like a polycrystal (λ = −62 ppm), whereas those with uniaxial orientation behave like a monocrystal (λ = −140 ppm). Correspondingly, the length of the crystalline phase will depend on whether poly- (Equation (30a)) or mono-crystalline (Equation (30b)) hcp-type nanoprecipitates are considered during the evaluation:

(30a) $$x_{cr}=0.75-\frac{2}{3}\frac{1}{62}\left(70-k_{\exp}\right)$$

or


(30b) $$x_{cr}=0.75-\frac{2}{3}\frac{1}{140}\left(157.5-k_{\exp}\right)$$

In Figure 6 and Figure 7, the stress-induced anisotropies and the corresponding record small permeabilities are presented. Figure 6 reveals an increase in stress sensitivity at induced anisotropies above 400 MPa. This would correspond to more monocrystalline (λ = −140 ppm) than polycrystalline (λ = −62 ppm) precipitates being produced at a higher stress level. Notably, while the stress sensitivity is unique for Fe-based MANCs (Ku = 11·σ), different composition-dependent, hardly predictable stress sensitivities were found [22,23,24,25] for Co-based MANCs, with Ku = 75·σ being found to be the maximal value so far. Comparing Figure 5 and Figure 7, both types of MANCs show hyperboloid-like µ_eff_ versus σ dependencies, with order-of-magnitude differences in the minimal effective permeabilities obtained for Fe-based (µ_eff_ = 140) and Co-based (µ_eff_ = 10) MANCs.

## 4. Evaluation of Linearity from Hysteresis Loop Measurements

**Figure 8:**
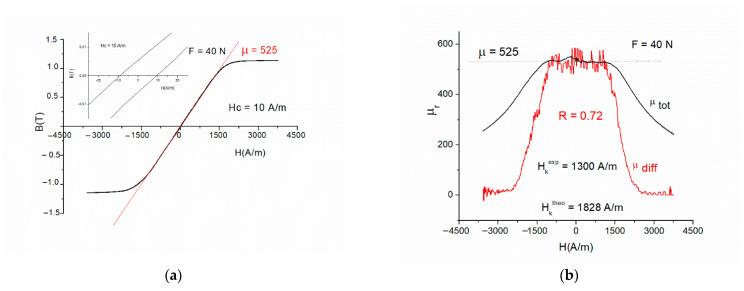
Linearity-related quasi-DC hysteresis loop characteristics. (**a**) Flatness (µ = 525) and negligible coercivity Hc = 10 A/m << Hsat = 2500 A/m. (**b**) The extent of linearity, Hlin, determined as the width of the permeabilities (µ_diff_ = dB/dH and µ_tot_ = B/H) plateau.

In Figure 8, a quasi-DC hysteresis loop of a Fe-based MANC sample is presented. One can delimit two regions only: the linear

B = µ_eff_·µ_o_·H if H < H_lin_ (31)

and the approach to the saturation part

B/Bs = 1 − a/H if H_lin_ < H < H_sat_ (32)

where *a* is the Neel constant, which represents the heterogeneities of free volume distributions whose internal stress fields produce a distribution of magneto-strictive anisotropies preventing alignment with saturation. The larger the parameter *a*, the more difficult it is to obtain the saturation and the more restricted the linear part.

The extent of linearity determines the experimental anisotropy field ${H_{K}}^{\exp}$, and the limit of flatness determines the theoretical anisotropy field: ${H_{K}}^{theo}=B_{s}/\mu \cdot \mu_{o}$. The ratio of these two fields is taken as the degree of linearity: $R=\frac{{H_{K}}^{\exp}}{{H_{K}}^{theo}}$

It is worth mentioning that Equation (32) can be taken as the first term of the expansion of the exponential:

(33) $$B=B_{s}\cdot \exp(-a/H{)}_{–}$$

One or two exponentials, corresponding to domain rotation (DR) and domain wall movement (DWM), can be used to simulate the magnetization region between H_lin_ and H_sat_.

**Figure 9:**
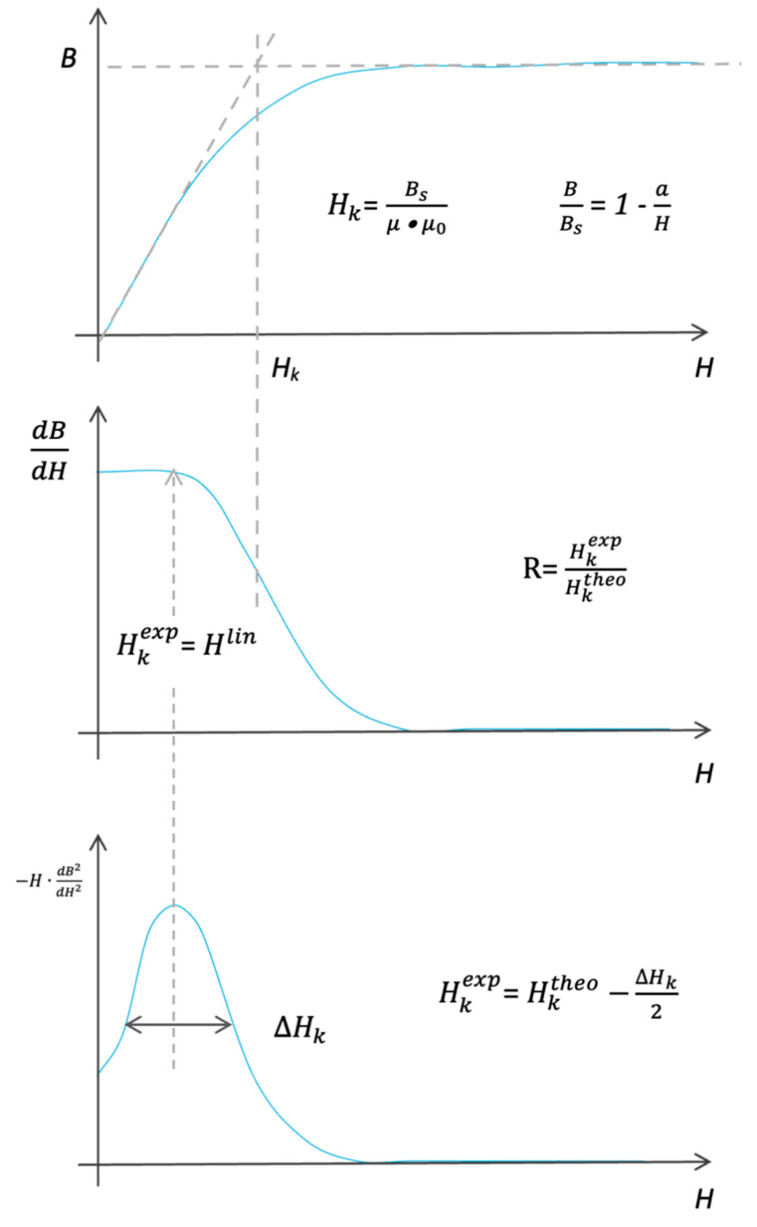
Schematic drawing of linearity-related DC hysteresis loop characteristics.

With the help of Figure 8 and Figure 9, the following linearity-related characteristics can be defined:

1. Anisotropy field calculated from effective permeability ${H_{K}}^{theo}=B_{s}/\mu \cdot \mu_{o}$.

2. Anisotropy field calculated from the second derivative [11,12] (note: noise increases with the order of derivation) or from fitting the first derivative with a relaxation-type function.

3. *p*(*H_K_*) is the distribution function of the anisotropy fields. It has a log-normal form, and D is its dispersion.

(34) $$p(H_{k})=-H\frac{d^{2}B}{dH^{2}}$$

4. H_lin_ = H_K_ − D, where D = (1 − x)·H_K_, such that

H_lin_ = x·H_K_ (35)

5. B_lin_ = x·B_s_; only precipitated particles participate in magneto-elastically induced anisotropy.

6. x can be determined as x = B_L_/Bs or x = H_L_/H_K_.

7. The degree of linearity is defined as

R = H_lin/_H_K_ =B_lin_/B_s_. (36)

8. The fraction of crystalline (bcc Fe_80_Si_20_ or hcp Co) phase is as follows:

x = R(37)

This is the most important result.

Observe that both the mean value, $H_{K}$, and its distribution, $\Delta H_{K}$, are linearly dependent on the applied stress:

*H_K_* = *C*_1·σ_ (38)

and

Δ*H_K_* = C_2_ + C_3·σ_ (39)

The stress dependence of the linearity degree can be expressed as

(40) $$R=\frac{H_{k}-\frac{1}{2}\Delta H_{k}}{H_{k}}$$

Inserting Equations (38) and (39) into Equation (40), one obtains

(41) $$R=1-\frac{c_{3}}{2c_{1}}-\frac{c_{2}}{2c_{1}}\frac{1}{\sigma }$$

The anisotropy field H_k_ can be expressed as a function of loop characteristics:

(42) $$H_{k}=\frac{3\lambda_{s}x_{cr}}{B_{s}}\cdot \sigma$$

For Fe-based MANCs

(43) $$H_{k}=17\cdot \sigma$$

For Co-based MANCs

(44) $$H_{k}=135.8\cdot \sigma$$

In the case of Fe-based MANCs, for the stress dependence of the distribution of the anisotropy field, one of the authors obtained the following:


(45) $$\Delta H_{k}=54+8.71\cdot \sigma$$

Using Equations (44) and (45), the degree of linearity for Finemet is as follows:


(46) $$R=0.74-\frac{1.58}{\sigma }$$

R is somewhat dependent on the applied stress; i.e., on the planned permeability. The degree of linearity depends solely on the time–temperature parameters of the nano-crystallizing heat treatment. The measure of linearity is equal to the fraction of the crystalline phase responsible for the induced anisotropy.

In the case of Co-based MANCs, the stress dependence of $\Delta H_{K}$ has not yet been elucidated.

## 5. Conclusions

The preparation and characterization of a flat-linear magnetization curve were presented using Fe- and Co-based nanocomposites.

In addition to the Finemet-type Fe-based metal-amorphous nanocomposite (MANC), the magnetic properties of the Co-based nanocomposite were investigated for the purpose of producing inductive cores with low permeability and a linear magnetization curve. The nanocomposites can be produced by heat-treating an amorphous ribbon of the appropriate composition under tensile mechanical stress. In terms of the achievable low permeabilities, the two nanocomposites complement each other: permeability values between µ = 3000 ÷ 100 can be achieved with the Fe-based MANC, while those between µ = 100 ÷ 10 can be achieved with the Co-based MANC. For the nanocomposite, an amorphous pre-alloy with a hypo-eutectoid composition is required, which is supplemented with a nucleation-promoting additive (1at% Cu) and a grain growth-inhibiting element (Nb). In the case of Co-based nanocomposites, the soft magnetic property is only preserved after nano-crystallization if we start from a so-called zero-lambda alloy, where the Co/Fe ratio is around 95/5. To the classic zero-lambda composition, Co_75_Fe_5_Nb_4_B_14_Si_2_, 2–5 at% early transition metals (Ti, V, Cr) are added to promote the formation of hcp Co.

The main findings of the present research are the following:

- In the case of Co-based MANCs, the induced anisotropy energy is one order of magnitude higher than that in Fe-based MANCs. The basic assumption of this work is that this results from the fact that the magneto-crystalline and magneto-strictive coefficients of hcp Co is one order of magnitude higher than those of the Fe_80_Si_20_ nanoprecipitate. A further basic assumption is that the Herzer back-stress model is valid for both Fe- and Co-based MANCs.
- Due to the two allotropic phases of Co nanoprecipitates, FCC and HCP, the induced anisotropy cannot be predicted or planned. However, because the induced anisotropy originates in hcp Co, the hcp fraction is equal to the degree of linearity, which can be determined based on the DC hysteresis curve.
- The heat treatment takes place in an open tubular furnace with a variable temperature gradient along the tube and variable holding time. The highest degree of linearity corresponds to the optimal heat treatment time–temperature parameters. Optionally, if deemed necessary, a supplementary long-term heat treatment at 330 °C can be conducted in order to promote the FCC-HCP transformation.

## Data Availability

The original contributions presented in this study are included in the article. Further inquiries can be directed to the corresponding author.
